# IgG4-mediated M2 macrophage polarization in tertiary lymphoid structures of esophageal cancer: implications for immunosuppression

**DOI:** 10.3389/fimmu.2024.1497783

**Published:** 2025-01-17

**Authors:** Hui Wang, Jirui Li, Yinghai Wang, Yang Chen, Weifeng Zhang, Xinyan Pan, Chanjuan Su, Ziteng Li, Li Wang, Jiang Gu

**Affiliations:** ^1^ Department of Pathology, The First People’s Hospital of Yunnan Province, Kunming, Yunnan, China; ^2^ The Affiliated Hospital of Kunming University of Science and Technology, Kunming, Yunnan, China; ^3^ Provincial Key Laboratory of Molecular Pathology and Personalized Medicine Center of Collaborative and Creative Center, Department of Pathology and Pathophysiology, Shantou University Medical College, Shantou, Guangdong, China; ^4^ Department of Gynecology, Peking University Cancer Hospital Yunnan, Yunnan Cancer Hospital, The Third Affiliated Hospital of Kunming Medical University, Kunming, China; ^5^ Jinxin Research Institute for Reproductive Medicine and Genetics, Xinan Hospital for Maternal and Child Health Care, Chengdu, China

**Keywords:** immunoglobulin G4, tertiary lymphoid structure, tumor microenvironment, macrophage polarization, interleukin-10

## Abstract

**Background:**

Our previous research highlighted the potential role of immunoglobulin G4 (IgG4) in mediating immunosuppression within the tumor microenvironment (TME). Tertiary lymphoid structures (TLS) in the TME have important immune-related functions. This study aims to analyze the distribution characteristics of IgG4-expressing cells, regulatory T cells (Tregs), and M2-type macrophages as well as to elucidate the relationship between IgG4 and the polarization of M2 macrophages within TLS in esophageal cancer.

**Object:**

To elucidate the distribution of IgG4, Treg cells, and M2 macrophages in TLS and to assess the impact of IgG4 on macrophage polarization.

**Methods:**

Esophageal cancer tissue were analyzed with multiplex immunofluorescence to determine the spatial distribution and density of B cells, T cells, and their subtypes. The relationship between IgG4 and CD8+ T cells in TLS, along with interleukin-10 (IL-10) expression and Treg presence, was studied. Serum IgG4 and IL-10 levels were compared between patients and healthy controls. *In vitro*, the impact of IgG4 on monocyte differentiation into M2 macrophages was observed.

**Results:**

IgG4 density was inversely related with CD8+ T cells in mature TLS indicating a potential immunosuppressive role (P<0.05,*). Serum analysis revealed that both IgG4 (P<0.01, **) and IL-10 (P<0.0001, ****) were significantly elevated and positively correlated in tumor patients compared to controls (P<0.01, **). *In vitro* experiments confirmed that IgG4 monocyte differentiation into M2 macrophages, potentially enhancing the immunosuppressive phenotype in TLS.

**Conclusion:**

IgG4 and IL-10 may contribute to immunosuppression in esophageal cancer by promoting the polarization of M2 macrophages within TLS, which could be a therapeutic target.

## 1 Introduction

TME is a complex ecosystem that emerges in response to tumor antigens, encompassing a diverse array of cellular and acellular components. These include vasculature, immune cells, fibroblasts, bone marrow-derived inflammatory cells, signaling molecules, and the extracellular matrix (1). Recent advancements in cancer immunology have categorized the TME based on the extent of immune cell infiltration, distinguishing between immune-infiltrating, immune-excluding, and immune-desert phenotypes (2–4). The TME’s immune landscape is shaped by the dynamic interplay between primary lymphoid organs, which generate immune cells, and secondary lymphoid organs, where these cells undergo maturation and activation.

Lymphocytes in the TME are mainly derived from the TLS, which is an important immune unit in the tumor tissues. TLS within the TME represent a critical adaptive immune component, morphologically akin to secondary lymphoid organs and functionally analogous in facilitating antigen-specific immune responses (5, 6). These structures, found inchronic inflammation, allograft rejection, and within neoplastic tissues, suggest a role in both immune surveillance and tumor progression. Recent attention has focused on tumor TLS and their presence within tumors as they influence the TME’s immunological profile and patient prognosis. Studies have correlated TLS density and immune cell infiltrates with improved clinical outcomes in various malignancies, including papillary thyroid, endometrial, and breast cancers (7–9). A close correlation has been found between survival and improved clinical outcomes in patients with solid tumors receiving cancer immunotherapy (10). Furthermore, a positive association between TLS presence and response to immunotherapy has been observed in soft tissue sarcoma, metastatic melanoma, and renal cancer (11–13). However, the dual nature of TLS in the TME is complex; while they can signify a favorable immunological context, they have also been implicated in tumor progression and evasion of immune surveillance. The signals involved in TLS production and the major cellular components of TLS have been extensively described, but their specific contributions to tumor immunity remain unclear (14). Despite the extensive characterization of TLS components and their signaling pathways, the precise contribution of individual TLS elements to tumor immunity remains an area of active investigation. This research aims to synthesize current knowledge on the formation, cellular constituents, and functional implications of TLS within the TME, highlighting their potential as therapeutic targets and biomarkers in cancer immunotherapy.

IgG, a pivotal humoral immune molecule synthesized by B cells, is pervasively present in the serum and extracellular fluids, exerting significant influence on both anti-infective and antitumor immunity. Our preceding research elucidated that IgG4 can modulate the antitumor efficacy mediated by other IgG subclasses through Fc-Fc engagements, potentially dampening immune surveillance against tumors (15). Specifically, IgG4 has been shown to indirectly modulate effector functions such as antibody-dependent cellular cytotoxicity (ADCC) (16), antibody-dependent cellular phagocytosis (ADCP) (17), and complement-dependent cytotoxicity (CDC) (18), thereby attenuating the immune system’s tumoricidal capabilities (19, 20). Moreover, the Fc region of IgG4 exhibits minimal affinity for Fcγ receptors and complement components, which can directly abridge the activation of the antitumor immune pathways, facilitating neoplastic progression (21, 22).

Recent research has advanced our knowledge of IgG4, confirming its antigen specificity and its ability to inhibit IgG1’s anti-tumor immune response. Nevertheless, the mechanisms underlying IgG4’s accumulation within TME, including the processes of IgG class switching, and the direct immunomodulatory effects of IgG4 on immune cells, remain unanswered puzzles. The elucidation of these mechanisms is crucial for a comprehensive understanding the immunoregulatory role of IgG4 in tumor biology and its potential as a therapeutic target in oncology.

TME is characterized by a Th2- dominant immune response, wherein the biased secretion of cytokines, including IL-4, IL-5, IL-10, and IL-13, contributes to immunosuppression. Notably, IL-10 within the TME, predominantly secreted by helper T cells and macrophages, has been implicated in the class-switching of B cells toward IgG4-producing plasma cells (23, 24). In 2012, Harada et al. observed that 43% of extrahepatic cholangiocarcinoma tissues exhibited IgG4 enrichment, suggesting that cholangiocarcinoma cells could function as non-professional antigen-presenting cells (APCs) and triggering IL-10-mediated IgG4 class switching, thereby augmenting the immunosuppressive milieu (25). Karagiannis et al. reported that 42.6% of melanomas, 21.4% of lymph nodes, and 30% of metastatic tissues exhibited heightened infiltration of IgG4-positive cells, concurrent with elevated serum IgG4 levels and an association with adverse prognosis (26, 27). Elevated IgG4 expression has also been detected in gastric cancer (28) and esophageal cancer (19), correlating with poor clinical outcomes. These findings identified the characteristic of IgG4 overexpression in malignant tumors, suggesting its potential role in facilitating tumor immune evasion.

This study focused on the distribution of immune cells, including CD8+ T cells, regulatory T cells (Tregs), and macrophages, within TLS. We aimed to understand the spatial distribution and potential sources of IgG4 within TLS and to assess the consequences of IgG4 interactions with immune cells on tumor immunosurveillance. The research aims to improve our knowledge of the interaction between IgG4 and the TME’s cellular components, providing insights into the mechanisms of the immunosuppressive phenotype and potential therapeutic strategies.

## 2 Materials and methods

### 2.1 Case selection and inclusion criteria

Serum samples from 33 healthy volunteers and 55 esophageal cancer patients were randomly selected. The inclusion criteria for healthy control participants encompassed individuals aged between 40 and 70 years, with no history of tumors or immune system disorders, and no recent administration of immunoglobulins or related blood products, among other factors. Serum samples from tumors were collected from either pre-treatment or post-treatment specimens of cancer patients, with the treatment modalities encompassing surgery, radiotherapy, and chemotherapy, while excluding immunotherapy. The patients’ age range was matched to that of the control group. Our study included 137 ESCC patients who underwent resection surgery and postoperative therapies at the Cancer Hospital of Shantou University Medical College from October 2013 to March 2017. The inclusion criteria of tissue samples can refer to our previously published articles. All samples obtained ethical and informed consent from relevant departments.

### 2.2 Immunohistochemistry and multicolor immunofluorescence

Immunohistochemical (IHC) and multiplex immunofluorescence (mIF) staining protocols were described in our previously published work. For IHC staining, paraffin-embedded tissue sections underwent dewaxing and rehydration before the antigen retrieval process. Subsequently, the sections were incubated with primary antibodies specific to the targeted immune cells, followed by incubation with the appropriate secondary antibodies. The stain-destain-restain (SDS) method was employed in TLS staining, whereby each cycle involved the removal of previously applied antibodies followed by a new round of staining. This process was iteratively conducted until all five antibodies—Ki67, CD21, CD20, CD4, and CD68—were applied. Specifically, Ki67 was used to identify proliferating lymphocytes, CD21 to visualize the follicular dendritic cell (FDC) network, CD20 to label a subset of B lymphocytes, CD4 to detect helper T cells, and CD68 to mark macrophages. IHC signals were developed using the AEC chromogenic substrate. For mIF, a sequential staining approach was employed. After the initial staining and coupling with fluorescent tags, antigen retrieval was performed. Subsequent to mIF, the procedure involved the conjugation and fixation of fluorescent dyes, antigen retrieval post-staining, and the elimination of preceding antibodies. This was followed by successive rounds of staining, culminating in the application of a total of five distinct staining antibodies. Following the completion of the staining procedure, the sections were analyzed utilizing a multispectral fluorescence microscope. Images were acquired, spectrally unmixed, and subjected to quantitative analysis to evaluate the spatial distribution and density of immune cell populations within TLS.

### 2.3 Monocyte sorting and polarization

Monocytes were isolated using the magnetic bead sorting technique. Venous blood samples were collected from healthy donors, and peripheral blood mononuclear cells (PBMCs) were subsequently obtained through density gradient centrifugation utilizing Ficoll-Paque (GE Healthcare). The PBMCs were resuspended in MACS Buffer, followed by cell counting using the Trypan blue exclusion method. CD14 magnetic beads (Miltenyi, 1×10^7^/20 μL beads) were added proportionally to the PBMC and incubated at 4°C for 15 min. Subsequently, CD14+ monocytes were selected by positive sorting. The matured adherent cells were collected after trypsin digestion.

### 2.4 Flow cytometry

Monocytes were cultured in 1640 complete medium supplemented with IgG1, IgG4, and a control group devoid of immunoglobulins, each at a protein concentration of 2mg/mL. After a period of seven days, flow cytometry analysis was conducted, focusing on the detection of IL-1β, INOS, IL-12, and CD163. Following the trypsinization of the cells, 5 µL of Human TruStain FcX (Fc receptor blocker) was added, and the mixture was incubated at room temperature for 10 minutes. Surface antigen detection involves the addition of specific flow cytometry antibodies targeting cell surface markers to each sample tube, followed by incubation in the dark at 4°C with continuous agitation for 30 minutes. Subsequently, 1mL of MACS buffer is added, and the samples are centrifuged and washed twice. For intracellular antigen detection, 5 µL of the appropriate intracellular flow cytometry antibodies are added, with incubation under similar conditions (in the dark at 4°C with shaking) for 15-30 minutes. Following this, 1 mL of Perm/Wash buffer is added, and the samples are centrifuged and washed twice. The supernatant is then removed, and the cells are resuspended in 200 µL of MACS buffer. Finally, flow cytometry analysis is conducted using the BD C6 flow cytometer.

### 2.5 ELISA assay

For the Serum IgG4 and IL-10 ELISA assays, wells were systematically prepared with standards, blanks, and appropriately diluted samples (400-fold dilution for IgG4 and 4-fold dilution for IL-10), each in a volume of 100 μL. The plates were subsequently incubated at 37°C for a duration of 90 minutes, with careful attention to minimize contact with the well walls and to prevent the formation of air bubbles. After aspiration and triple washing, 100 μL of biotinylated antibody was added, followed by a 60-minute incubation at 37°C. Wells were drained and washed thrice with 300 μL of buffer. Subsequently, 100 μL of HRP-conjugated enzyme was added and incubated for 30 minutes at 37°C. Following another triple wash, 100 μL of TMB substrate was added and incubated for 15 minutes, with color development monitored to prevent over 30 minutes. The reaction was terminated with 50 μL of stop solution and optical density measurements at 450 nm were conducted within a 5-minute timeframe.

### 2.6 Statistical method

All data were analyzed by GraphPad Prism 9.0 and SPSS software. Significant differences between different groups were assessed by Student’s t test, ANOVA multiple comparison tests, non-parametric testing, and simple linear regression. Statistically significant differences were labeled as: ns, no significance (p < 0.05 was considered statistic significant), *P < 0.05, **P < 0.01, ***P < 0.001.

## 3 Results

### 3.1 Structural characteristics and classification of TLS

**Figure 1:**
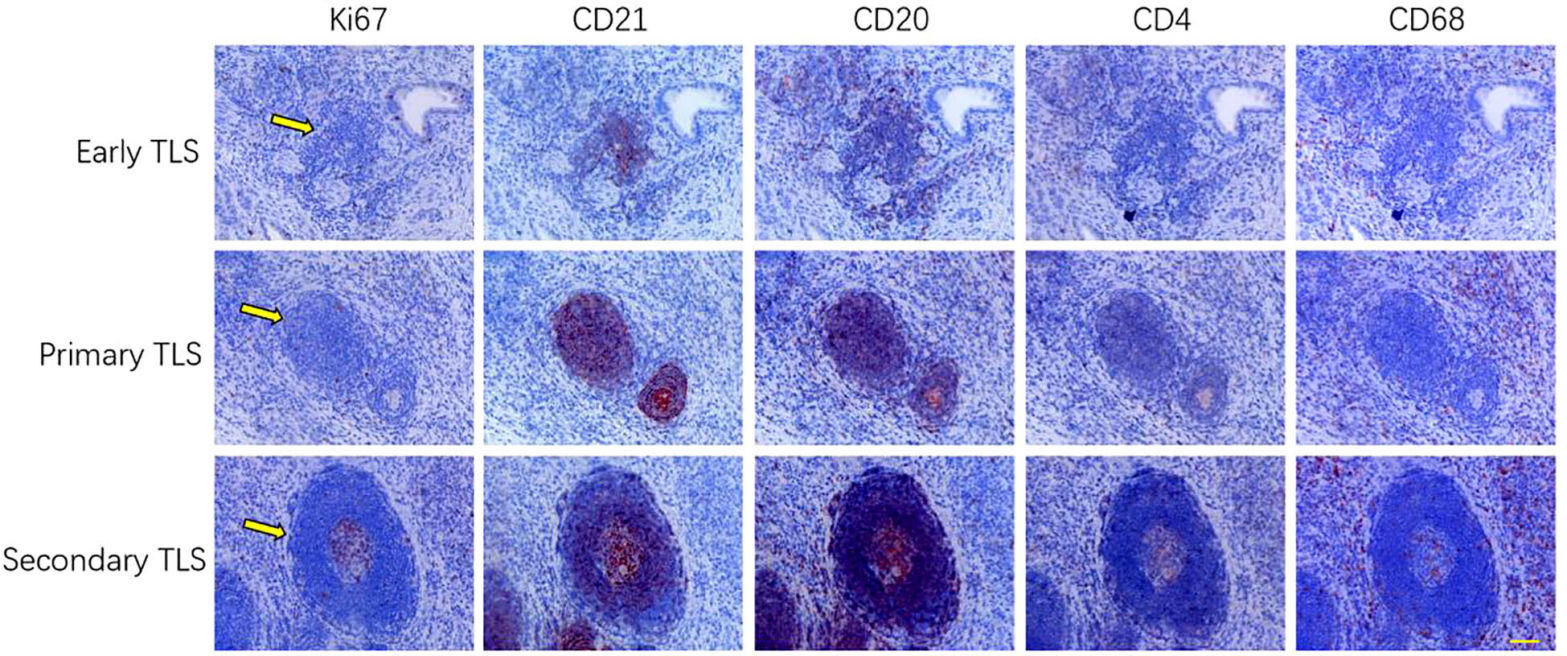
The classification of TLS is determined by several key indicators, categorizing them into three distinct stages: early, primary, and secondary TLS. This classification is based on the density of lymphocytes, the propensity for follicular structure formation, and the development ofGCs (n=3). Scale bar=60um.

In this study, TLS were classified into three distinct types—early TLS, primary TLS, and secondary TLS—based on the degree of lymphocyte aggregation and the proliferative status of B cells and dendritic cells within the follicular centers (**Figure 1**). This classification utilized five specific markers: Ki67, CD21, CD20, CD4, and CD68. The early TLS are characterized by a relatively clustered arrangement of B cells and FDCs. Consequently, primary TLS exhibit pronounced elliptical lymphoid structures. In contrast, secondary TLS undergo further development, resulting in the formation of distinct T/B cell zones and follicular germinal centers (GCs). Additionally, the elevated expression of nuclear proliferation marker ki67 in follicular center indicates the maturation and functional optimization of secondary TLS.

### 3.2 Expression of IgG4 in TLS and characterization of T cells

**Figure 2:**
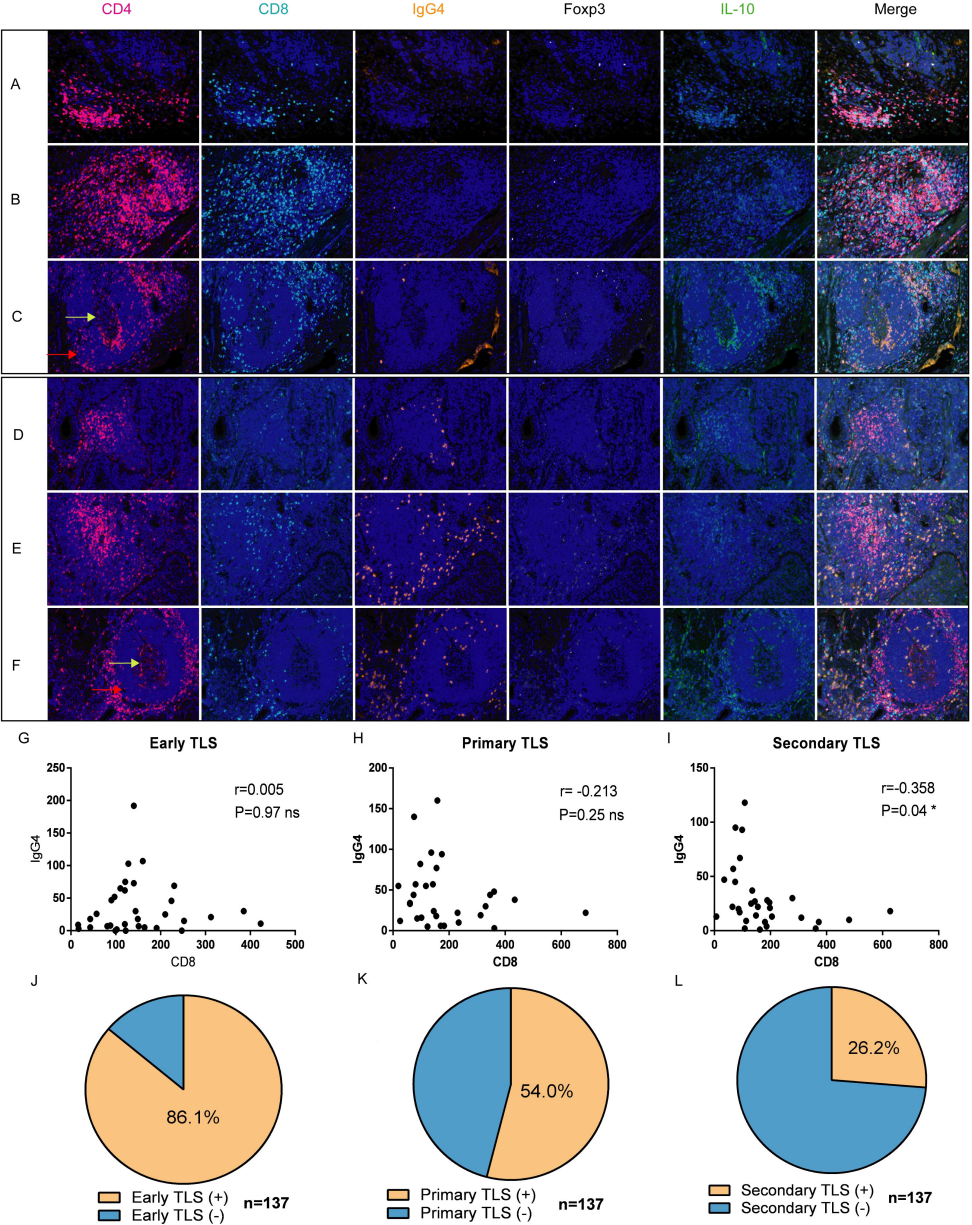
Expression and distribution of IgG4 with CD4, CD8, Foxp3+, and IL-10+ cells in TLS of different developmental states [n=137, **(A, D)** early, **(B, E)** primary, **(C, F)** secondary]. **(G–I)** correlation analysis of CD8+ T cells and IgG4-associated plasma cells in TLS at three different developmental stages (Early TLS n=33, Primary TLS n=30, Secondary TLS n=31). **(J–L)** respectively demonstrate the positive proportions of three types of TLS (total n=137).

Multiple fluorescent staining techniques was employed to identify various immune markers within tumor tissues (**Figure 2**, n=137). Our findings indicate that the abundance of CD8+ T cells in the three cohorts of TLS exhibiting elevated IgG4 expression is markedly reduced in comparison to the cohort of TLS with diminished IgG4 expression (**Figures 2A–F**). In mature TLS, the number of CD8 and IgG4 cells exhibits an inverse relationship, which is statistically significant (**Figure 2I**, r=-0.358, P=0.04 *). This result suggests that within the tumor immune microenvironment of esophageal cancer, cytotoxic immune responses represented by CD8 and B cell-mediated tumor immune suppression characterized by IgG4 may influence disease progression in two opposing directions. This also underscores the complexity and paradoxical nature of the tumor microenvironment. In the 137 esophageal cancer samples, the positive rate of early TLS was 86.1%, the positive rate of primary TLS was 54%, and the positive rate of mature secondary TLS was 26.2% (**Figures 2J, K, L**). Additionally, mature plasma cells expressing IgG4 were present in all three different states of TLS, indicating that mature B cells were present in both mature and immature TLS. This suggests that the microenvironment contains plasma cells from multiple sources, which may not strictly align with the developmental pattern of B cell maturation and differentiation in the follicular center.

### 3.3 IgG4 and IL-10 levels in the serum of cancer patients were significantly higher than those in healthy individuals

**Figure 3:**
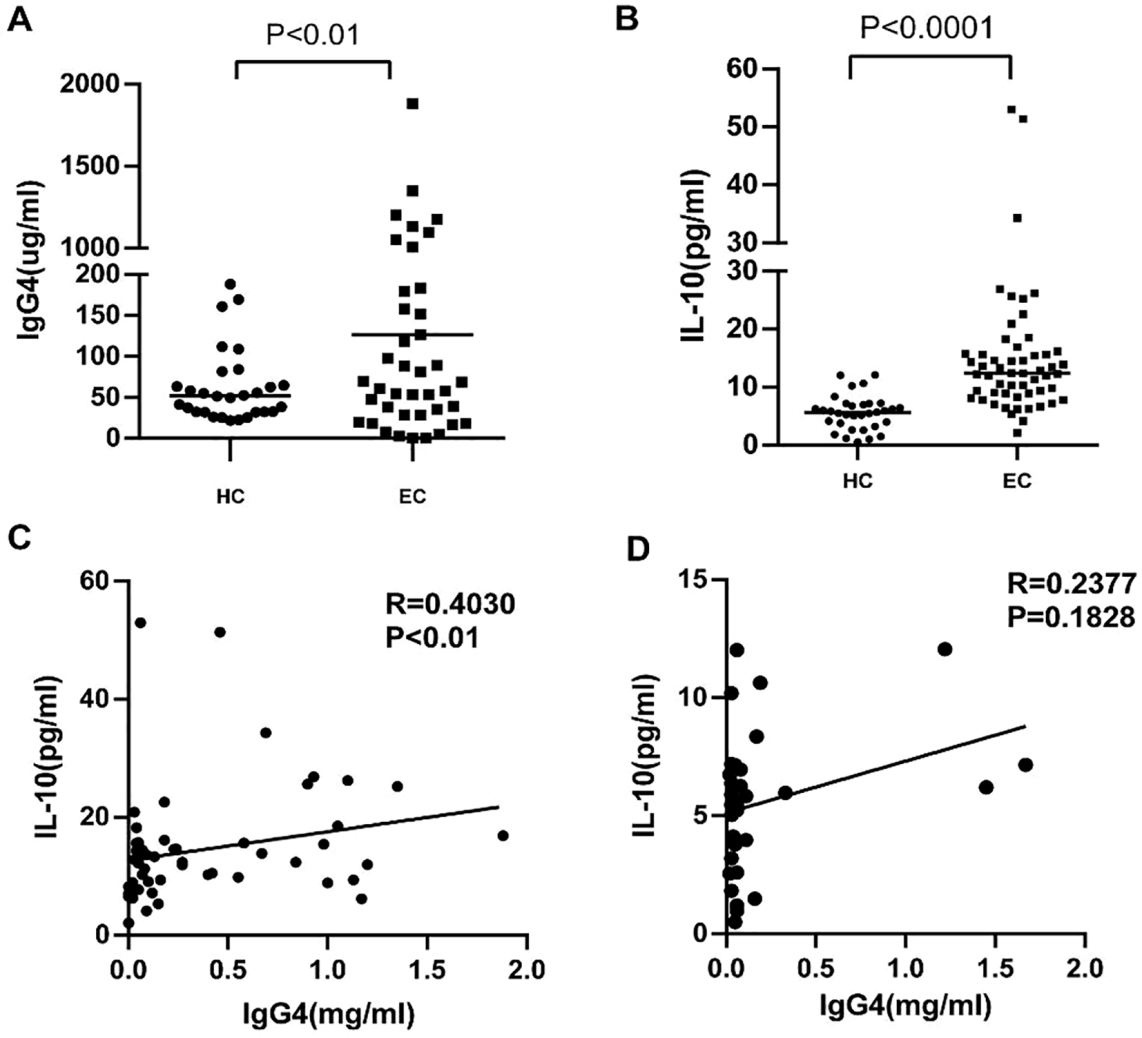
**(A, B)** Serum IgG4 and IL-10 levels in esophageal cancer patients (EC) and Healthy controls (HC). **(C, D)** The correlation analysis between serum lgG4 and IL-10 concentration in the serum of EC (n=55) and HC (n=33).

In our prior investigations, we extensively analyzed the expression of IgG4 and IL-10 within TLS and peripheral mesenchyme. In the present study we extended our examination to include the serum of tumor patients and healthy controls assessing levels of IgG4 and IL-10. The findings revealed positive correlation between these biomarkers, with the association being particularly pronounced in the cohort of cancer patients. Serum samples were randomly selected from 33 healthy volunteers and 55 esophageal cancer patients aged 50 to 70 years old. As shown in **Figures 3A, B**, the levels of IgG4 (P<0.01, **, **Figure 3A**) and IL-10 (P<0.0001, ****, **Figure 3B**) in the serum of esophageal cancer patients were significantly elevated compared to those in healthy controls. IgG4 and IL-10 are anti-inflammatory cytokines known to suppress inflammatory responses and inhibit the activation of immune cells. The increased levels of IgG4 and IL-10 in esophageal cancer patients may be due to the inflammatory response and the subsequent increase in leukocytes within the body. The correlation between IgG4 and IL-10 in the serum of esophageal cancer patients, revealing a positive correlation (R=0.4030, P<0.01, **Figure 3C**) compared to healthy controls (**Figure 3D**). This could be attributed to the inflammatory responses present in esophageal cancer patients, which stimulate the production of IgG4 and IL-10. Higher levels of IL-10 indicate a more intense inflammatory response.

### 3.4 Expression of IgG4 and characterization of macrophages in TLS

**Figure 4:**
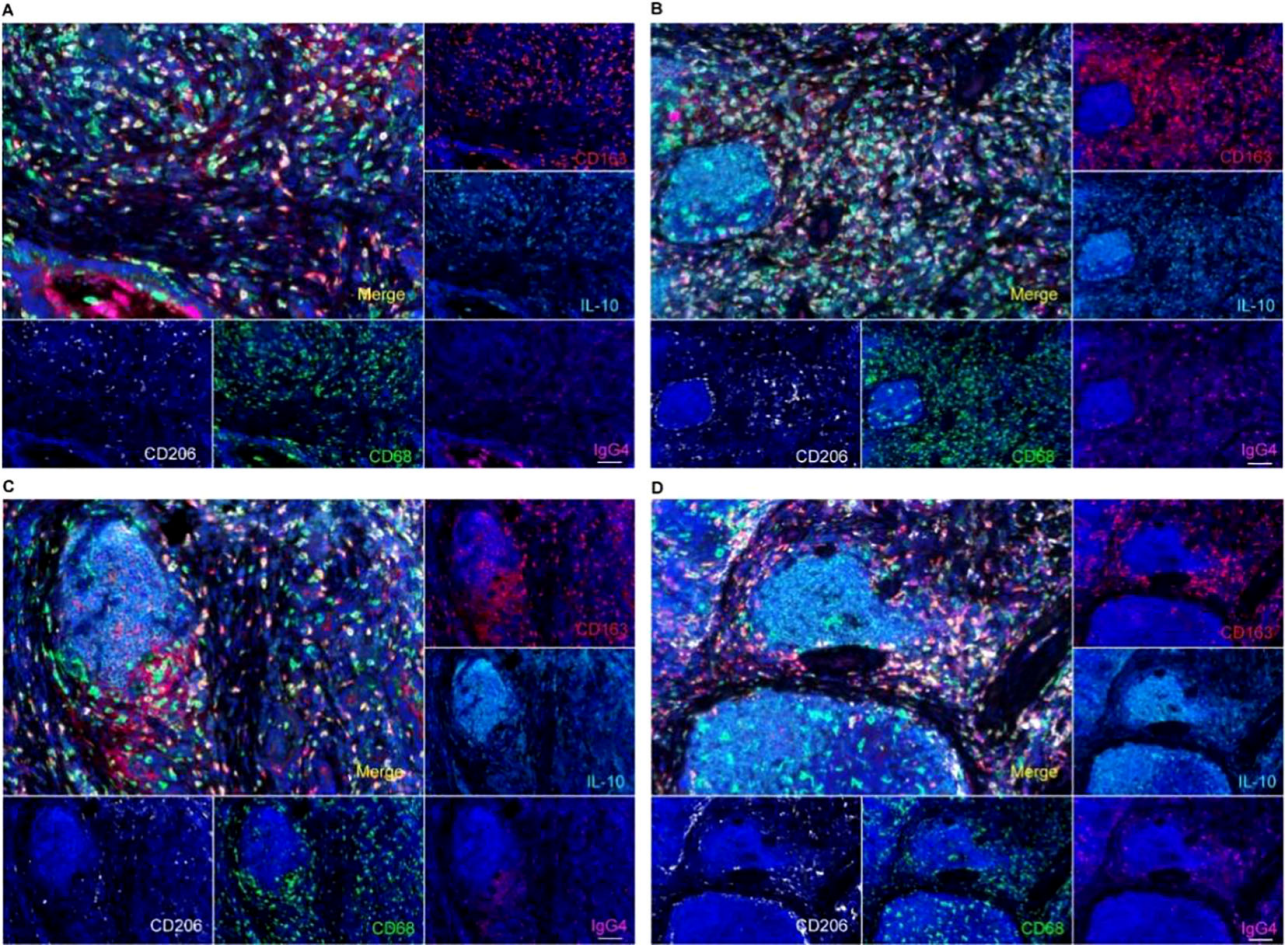
IgG4 and macrophage polarization phenotype analysis by multiple fluorescence staining in TLS, suggested a CD163+CD206+M2-type macrophages predominant tumor microenvironment in TLS (**A** tumor stromal macrophage aggregation zone, **B, C** primary TLS, **D** primary TLS and secondary TLS). Scale bar=60um.

Macrophages in TLS and the adjacent mesenchymal tissue predominantly exhibited an M2 phenotype, characterized by the expression of markers such as CD163 and CD206. Additionally, we observed elevated levels of IL-10 in the microenvironment, which correlated with a high density of M2 macrophages (**Figure 4**). Numerous studies have indicated a correlation between IL-10 and IgG4. It is therefore important to investigate the impact of elevated IgG4 levels on macrophages within the surrounding microenvironment.

### 3.5 High levels of IgG4 promote macrophage M2 polarization

**Figure 5:**
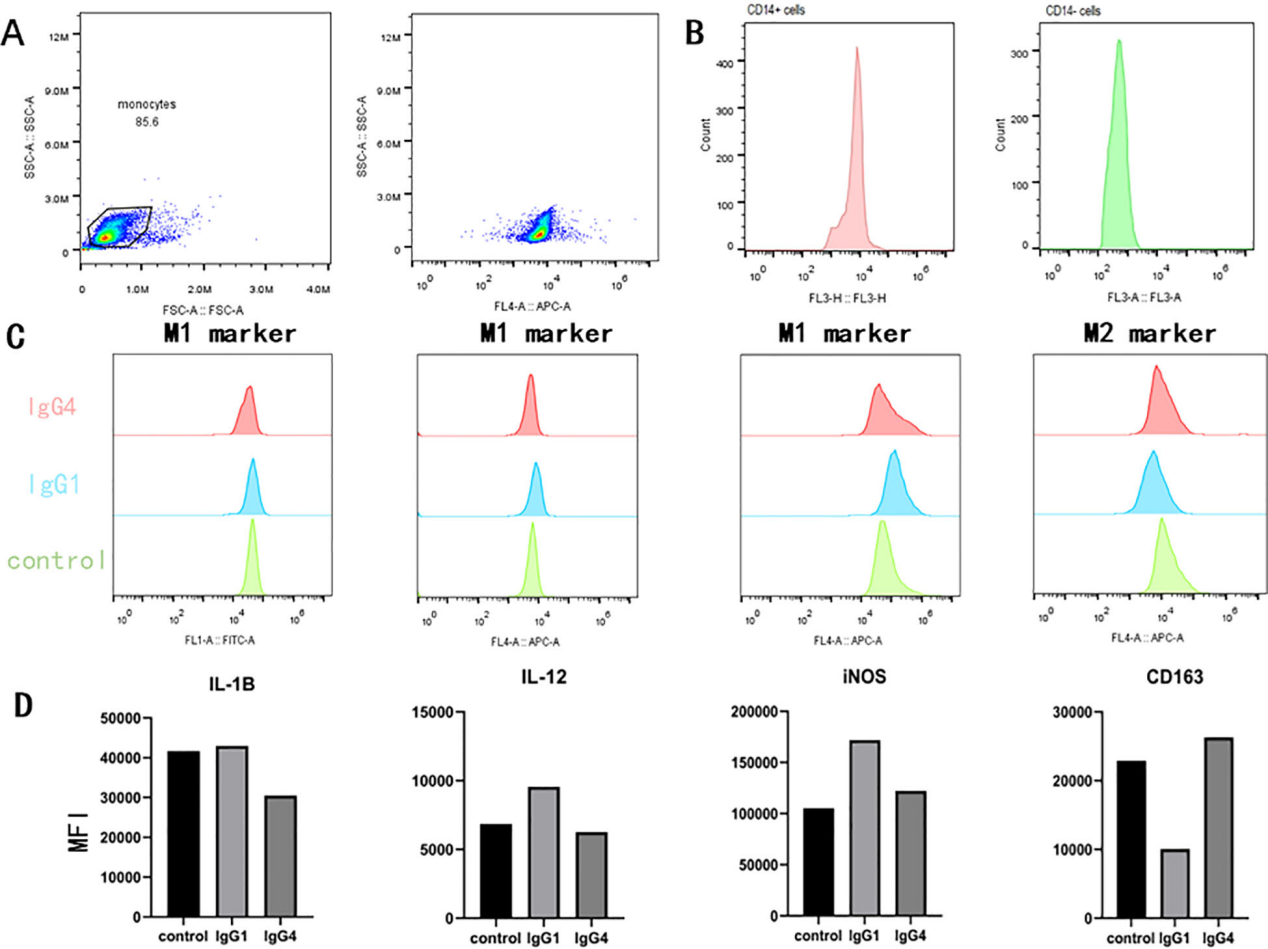
In an *in vitro* monocyte differentiation culture subjected to stimuli (HSA, IgG1, IgG4), IgG4 has been observed to promote the differentiation of monocytes into M2-type macrophage. **(A, B)** Validation of flow cytometry gating and CD14 magnetic bead sorting for positive cell potency. **(C, D)** Comparison of flow cytometry detection and mean fluorescence intensity after IgG1 and IgG4 stimulation of monocytes.

In the subsequent *in vitro* experiments, we aim to further elucidate the direct impact of IgG4 subtypes on monocyte differentiation (**Figure 5**). *In vitro* culture experiments, distinct morphological differences were observed in macrophages following stimulation with various proteins. Specifically, macrophages in the IgG4-stimulated group were predominantly exhibited an elongated spindle-shaped morphology, whereas those in the IgG1-stimulated group predominantly displayed an oval shape. Macrophages stimulated by IgG4 demonstrated elevated expression levels of M2-type macrophage markers (CD163), while the IgG1 group exhibited higher expression levels of M1-type marker (IL-1β, IL-12, iNOS) in subsequent flow cytometry analysis. The results of the *in vitro* experiment suggest that IgG4 has the potential to promote the differentiation of monocytes into M2 macrophages.

## 4 Discussion

TLS, characterized by the complex composition of immune cells and robust immune functionality, serve as the primary site for anti-tumor immune responses within tumors. Research indicates that in the context of cancer immunotherapy for various solid tumors, such as esophageal cancer, bladder cancer, head and neck squamous cell carcinoma and pancreatic ductal adenocarcinoma, the maturity of TLS is significantly associated with improved prognostic outcomes (11–13, 29). In a large-scale cohort study on esophageal cancer TLS (394 and 256 ESCC patients from Sun Yat sen University Cancer Center and the Cancer Hospital of Shantou University Medical College, it was found that mature TLS was an independent prognostic factor for patients, and mature TLS with high levels of CD8 infiltration had a better prognosis (30). This study was limited by sample size and did not conduct statistical analysis between TLS and patient prognosis. Although mature TLS exhibits strong anti-tumor immune activity in cellular and humoral immune responses, the exact mechanism controlling its formation and functional pathways remains elusive. The presence of mature TLSs within tumor tissues is about 26.2% which is consistent with the spectrum of tumor-immune infiltration patterns. Tumor immune infiltration status can be categorized as immune-infiltrating, immune-excluding, and immune-desert (4, 5). These classifications are essential for predicting therapeutic responsiveness and customizing immunotherapeutic strategies. TLS serve as critical subunit for both cellular and humoral immune responses within the TME, underscoring the imperative for ongoing investigation into their role and dynamics. This study addresses two critical aspects: the conditions that may lead to IgG4 accumulation within TLS and the subsequent impact of IgG4 on macrophage polarization. The emergence of IgG4 within TLS is posited to be associated with elevated IL-10 expression by T helper cells and macrophages within the TME. We have described in detail in our previous study that IgG4 as a class of immunosuppressive proteins combined with the specificity of its structure can inhibit anti-tumor responses through the Fc-Fc effect to reduce ADCC, ADCP, CDC immunity and inhibiting anti-tumor response mediated by IgG1.

IL-10, a cytokine known for its anti-inflammatory properties, has been extensively studied for its role in fostering an immunosuppressive microenvironment. While the inhibitory effects of IL-10 on inflammation are well-documented, the precise mechanisms by which IL-10 modulates B cell class switching to IgG4 were reported in recent years (31). Future experimental work is necessary to dissect the intricate signaling pathways through which IL-10 influences B cell differentiation and the broader implications for tumor immune evasion. Understanding these mechanisms is essential for developing targeted therapies that could potentially disrupt the immunosuppressive activities of IgG4 and enhance the efficacy of immunotherapies. In a study on IgG4RD, researchers found that the number of cTfr cells producing IL-10 was significantly increased in IgG4RD patients compared to healthy elderly subjects, suggesting a possible correlation between IL-10 and IgG4 elevation, which is similar to our research findings (32). CD4+FOXP+IL-10+Regulatory T cells (Treg) serve critical roles in modulating immune effector cell activity, preventing tissue damage, and suppressing inflammatory responses. When exposed to tumor antigens and inflammatory cytokines, Treg cells undergo reprogramming that amplifies their immunosuppressive functions, thereby facilitating tumor immune evasion and contributing to tumor progression (33). our study provides compelling evidence that IgG4 plays a significant role in the immunosuppressive network within the TME of esophageal cancer. However, research on eosinophilic esophagitis suggests no significant association between IgG4 and IL-10 positive cells and pathological changes (34). In this study, the serum levels of IgG4 and IL-10 were detected and statistically analyzed. However, the relationship between the positive cell counts in tissues and the disease requires further refinement. The presence of TLS in a substantial proportion of tumor tissues underscores their potential as a therapeutic target. Our findings reveal a negative correlation between IgG4 density and CD8+ T cells within TLS, particularly in the context of high IL-10 expression, suggesting a mechanism by which IgG4 may modulate antitumor immune responses. Furthermore, the decrease in serum IgG4 and IL-10 levels following treatment implies a potential role for these markers in monitoring therapeutic efficacy.

Macrophage polarization has received increasing attention in the field of tumor immunity in recent years, and the *in vitro* culture and differentiation methods of macrophages are mature experimental procedures (35). Different cytokine stimuli can differentiate monocytes into macrophages of different phenotypes, but it is worth noting that there is currently little attention paid to the effect of immunoglobulin on macrophage polarization. Our experiment has a certain degree of originality. We used natural IgG protein derived from human serum to culture macrophages *in vitro* and found a unique effect on macrophages. This feature suggests the strong plasticity of macrophages and the regulatory ability of immunoglobulins. We found that this phenomenon is cell dependent during the experiment, with polarization characteristics being statistically significant in some individuals, while others are not. The reason we are considering here is that monocytes have certain individual differences in surface receptor types and abundance. On the other hand, considering the consistency of cell surface receptors, the binding affinity between antibodies and receptors also varies among individuals, which can lead to experimental failure (36). We will continue to investigate the underlying reasons for these individual differences in future experiments and hope to answer these questions. The polarization of macrophages toward an M2 phenotype under the influence of IgG4 presents a novel mechanism by which immunosuppression may be facilitated in esophageal cancer. This process, which is characterized by the upregulation of M2-type macrophage markers, has implications for the progression of the disease and the efficacy of immunotherapies. Our results highlight the complexity of the TME and the multifaceted role of IgG4 in shaping the immunological landscape. The identification of IgG4 as a key mediator of M2 macrophage polarization within TLS offers new avenues for therapeutic intervention. Future research should focus on validating the prognostic value of IgG4 and exploring the potential of targeting the IgG4-mediated immunosuppressive pathway as a strategy to enhance the effectiveness of immunotherapies in esophageal cancer.

TLS refers to the ectopic aggregation of immune cells and the subsequent initiation of immune responses within TME. CXCL13, produced by T follicular helper (Tfh) cells, directs the migration of CXCR5-positive B lymphocytes to areas of chronic inflammation, thereby promoting the formation of TLS and GCs. Under the influence of CXCL13, an initial chemotactic factor, lymphocytes migrate toward the target site and undergo continuous proliferation, characterizing a dynamic developmental process (37–39). Furthermore, chronically activated CD8+ T cells exposed to fibroblast-derived TGF-β secrete the B cell chemokine CXCL13 (37, 40, 41). This chemokine has been demonstrated to enhance the spatial distribution of B cells and facilitate the maturation of tertiary lymphoid structures (TLS). Numerous studies have investigated tertiary lymphoid structures (TLS), yet a consensus on their maturity classification remains elusive. The prevailing perspective suggests that early TLS is marked by the dispersed aggregation of B and T cells, with CD21 and CD23 being negative. In contrast, primary TLS is identified by the aggregation of B and T lymphocytes, with CD21 and CD23 being positive, but lacking plasma cells. Mature TLS, building upon primary TLS, is distinguished by the presence of GCs within lymphoid follicles and the emergence of plasma cells (42). Morphologically, the maturation of TLS is primarily characterized by the development of GCs, which signify active anti-tumor immune responses. Through the application of imaging mass cytometry, both TLS and secondary lymphoid organs (SLO) were analyzed, confirming the applicability of existing grading systems (43). Interestingly, in our observations, IgG4 plasma cells appeared in all three types of TLS, suggesting that their presence is not exclusively associated with mature TLS. Given that IgG4 is the least abundant subclass of IgG, it is plausible that other IgG subclasses may also exhibit elevated levels of expression. Given this context, the presence of plasma cells may not serve as a definitive characteristic of mature TLS. Furthermore, the morphological maturity of TLS may not be the primary determinant of patient survival. TLS are dynamic entities, and the specific cell types and their activation pathways within TLS are crucial for tumor immune regulation.

## 5 Conclusion

**Figure 6:**
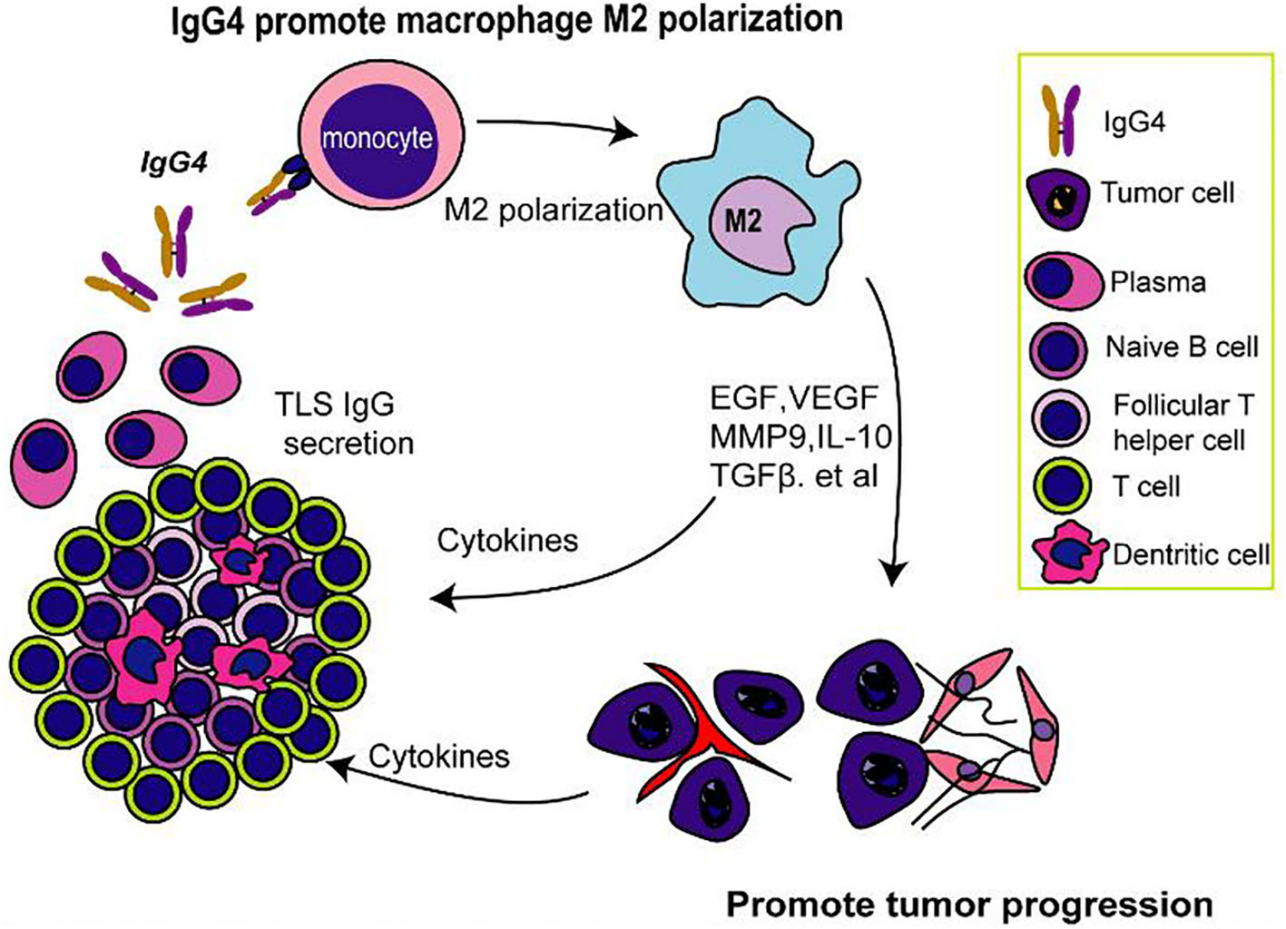
IgG4-mediated M2 macrophage polarization in TLS and its possible implications for immunosuppression.

Overall, our study contributes to the growing evidence that implicates IgG4 in the pathogenesis of esophageal cancer and emphasizes the need for a deeper understanding of the immunological mechanisms within the TME. This study identifies IgG4 as an important factor in the immunosuppression of esophageal cancer tumor microenvironment, particularly within TLS as illustrated in **Figure 6**. The negative correlation between IgG4 and CD8+ T cells, along with the association between IgG4 and M2 macrophage polarization, reveals a potential mechanism for immune evasion in tumors. The modulation of macrophage polarization by IgG1 and IgG4 may have significant implications for cancer progression and the response to immunotherapy. Our findings underscore the importance of further research into the role of IgG4 in cancer immunity, with the aim of developing targeted therapies to counteract the immunosuppressive effects and improve therapeutic approaches and clinical outcomes.

## Data Availability

Data reported in this article will be available upon reasonable request. Requests to access the datasets should be directed to HW, 997356768@qq.com.
